# 

**Published:** 1888-02

**Authors:** 


### CONTENTS, FEBRUARY, 1888, (VOL. III, NO. VIII.)

ORIGINAL ARTICLES.—The Treatment of Dengue, Cholera Infantum, etc.: by Dr. T. J. Heard, Galveston....... 309
Removal of a Shot from the Eye Six Months after Injury: by Dr. Wm. Caston, Corsicana ................ 311
Pleurodynia: by Dr. R. P. Davies, Petty.............. 314
A Novel Theory in Regard to an Additional Function of the Liver: by Dr. F. A. Schmitt, Schulenburg........ 318
Treatment of Acute Pulmonic Inflammations in Young Children: by Dr. Q. C. Smith. Austin............... 321
CORRESPONDENCE.—"Cholera in New York" Criticised; The Quarantine Question: letter from Dr. A. S. Wolff, Brownsville.................................... 325
Dysentery............................................ 338
SOCIETY NOTES.—Austin District Medical Society....... 325
Proceedings of Terrell Medical Society.............. 340
CULLINGS FROM CONTEMPORARIES.—The Choice of General Anæsthetics in Surgery and Obstetrics......... 343
EDITORIAL.—The Coming Meeting at Galveston......... 354
Dr. Wolff's Criticism.............................. 355
Physicians' Mutual Benefit Association............... 356
MEDICAL NEWS AND MISCELLANY....................... 358
ADVERTISERS' NOTICES................................ 359

